# Assessing the diagnostic performance of clinical, serological and molecular approaches to improve dengue case detection in the Peruvian Amazon

**DOI:** 10.1371/journal.pntd.0013984

**Published:** 2026-02-09

**Authors:** Anne Hauner, Jaide Aroni-Sierra, Xiomara Merino, Carlos Villa, Fiorella Torres, Ole Lagatie, Michael Talledo, Kevin K. Ariën, Francesca Falconi-Agapito

**Affiliations:** 1 Department of Biomedical Sciences, Institute of Tropical Medicine, Antwerp, Belgium; 2 Instituto de Medicina Tropical Alexander von Humboldt, Universidad Peruana Cayetano Heredia, Lima, Peru; 3 Laboratorio HSG, Hospital Santa Gema, Yurimaguas, Peru; 4 Oficina de Epidemiología, Hospital Santa Gema, Yurimaguas, Peru; 5 Department of Communicable Diseases, Johnson and Johnson, Beerse, Belgium; 6 Department of Biomedical Sciences, University of Antwerp, Antwerp, Belgium; University of Hawaii at Manoa, UNITED STATES OF AMERICA

## Abstract

In dengue endemic, resource-limited settings, accurate and timely diagnosis is critical for effective clinical management and outbreak control, especially where multiple arboviruses co-circulate and overlap in clinical presentations. However, most dengue diagnosis in such settings rely on approaches with limited sensitivity such as clinical assessment, or easily deployable methods such as ELISA or rapid diagnostic tests (RDTs). Molecular diagnostics with superior diagnostic performance are rarely implemented beyond reference laboratories due to perceived logistical and operational barriers. This study provides real-world evidence comparing the performance of clinical, serological and molecular approaches for dengue diagnosis in a decentralized setting. We prospectively enrolled 271 patients with acute febrile illness at Santa Gema Hospital in Yurimaguas, Peru, during a dengue outbreak in 2023–2024. Patients underwent clinical evaluation (WHO 2009 dengue classification), and laboratory testing including NS1/ IgM RDTs and ELISAs, a triplex RT-PCR for ZIKV/DENV/CHIKV (ZDC-PCR), a newly developed multiplex RT-PCR for ZIKV/YFV/DENV/CHIKV (ZYDC-PCR), and a serotype-specific dengue RT-PCR used as reference. Diagnostic performance was assessed using sensitivity, specificity, ROC-AUC analysis, and logistic regression models. A subset of 131 samples underwent inter-laboratory comparison of the ZYDC-PCR between the regional (Yurimaguas) and central (Lima) laboratories. Of the 271 dengue-suspected cases, 88 (32.6%) were confirmed by the reference PCR. The ZYDC-PCR had a strong agreement with the reference (sensitivity 86.0%, Cohen’s kappa 0.893) and consistent performance across the central and regional laboratory. NS1-based tests showed high specificity (≥96%) but moderate sensitivity (~72%). ROC analysis confirmed the accuracy of PCR (AUC = 0.97), outperforming RDTs, ELISAs (AUC = 0.85 to 0.89) and clinical assessment (AUC = 0.65). Our study demonstrates the added value and feasibility of implementing a multiplex PCR at a regional hospital to significantly improve diagnostic accuracy, enabling earlier detection of disease presence or absence, critical for clinical management and outbreak response.

## Author summary

Dengue is a viral infection spread by *Aedes* mosquitos that causes fever and, in some cases, can lead to severe illness. For proper patient care and outbreak control, timely and accurate case confirmation is essential. However, in many dengue endemic areas, especially in remote and low-resource settings, access to reliable laboratory tests is limited. As a result, dengue suspected cases are often diagnosed based on clinical symptoms alone or simple rapid tests, that may miss true infections. In this study, carried out in a district hospital in the Peruvian Amazon, we compared how well different diagnostic approaches work in real-world conditions. We evaluated: (1) diagnosis based on symptoms, (2) rapid tests and ELISA tests that detect the dengue protein NS1 or antibodies and (3) three different PCR tests that detect the virus’s genetic material. One of the PCRs was a new test detecting simultaneously dengue and three other arboviruses. We found that adding NS1 rapid tests or NS1 ELISA to clinical diagnosis improved dengue detection, but these tests still missed many confirmed infections. PCR testing showed highest accuracy, and the new PCR worked similar to the reference test, even when used in a small district hospital setting. Despites the encountered logistic, infrastructure and trained human resources challenges, our findings show the feasibility of PCR decentralization in a district hospital in the Peruvian Amazon.

## 1. Introduction

In low- and middle-income countries (LMICs) fever is a frequent reason for seeking medical care. In the case of no evident localized focus of infection, these cases are classified as acute undifferentiated febrile illness (AUFI). AUFIs typically present with non-specific symptoms including chills, headache and myalgia, but with the progression of the illness, more specific organs can be involved. They are broadly categorized as malarial and non-malarial AUFIs, caused by viral, bacterial or eukaryotic pathogens [1,2].

Malaria remains a major cause of acute febrile illness in the Peruvian Amazon. Although the Malaria Zero elimination program significantly reduced the reported cases in Loreto to ~50%, prior to the COVID-19 pandemic [3], its incidence has increased again in several areas. Nevertheless, non-malaria causes of fever continue to represent a significant proportion of acute febrile illnesses and require diagnostic approaches that can accurately detect dengue and other co-circulating arboviruses.

Among non-malarial causes, arboviral infections, particularly dengue virus (DENV), are recognized as major contributions to AUFI in tropical regions [2]. In South America more than 30% of AUFI are caused by arboviruses, mostly by DENV [4]. In Peru, this proportion varies across regions, ranging from 20 to 50% [5–7]. The Amazon basin faces an especially complex epidemiological landscape, with frequent co-circulation of multiple arboviruses including flaviviruses (DENV, yellow fever virus [YFV], Zika virus [ZIKV]), alphaviruses (chikungunya [CHIKV], Mayaro [8,9], Venezuelan equine encephalitis virus [4,10–13]), and, most recently, the *Orthobunyavirus* Oropouche, [11,14–16].

To support outbreak response, the Peruvian Ministry of Health (MoH) implemented a technical standard in 2016 to strengthen arboviral surveillance, recommending virus isolation, real-time RT-PCR, NS1 antigen ELISA (for flaviviruses) and IgM ELISA as confirmatory tools [17]. Although the percentage of laboratory-confirmed cases for DENV nationwide, based on results from either regional laboratories or the national reference laboratory in Lima, has improved in recent years, confirmation remains distributed unevenly cross the country [18]. Whereas coastal cities such as Piura, have consistently high confirmation rates (>89% in recent years), Amazonian regions such as Loreto and Ucayali report substantially lower rates (around 50% in 2024) [17], reflecting diagnostic inequities across the regions most affected by arboviruses.

Molecular diagnostics remain particularly underutilized. Between 2018 and 2019, only 20% of confirmed dengue cases were diagnosed by RT-PCR, while ELISA was used for 77% (19% for IgM and 68% for NS1) [19]. While 19 out of 25 laboratories can test for DENV, only seven can diagnose YFV and testing for CHIKV and ZIKV remains centralized in Lima [20]. Public information on the specific methods used across laboratories is also limited.

Despite recent improvements in national confirmation rates, reliance on serological tools, long delays in reporting (up to 28 days post-sample collection allowed) [21] and the limited decentralization of molecular assays further limit the clinical utility of laboratory confirmation and compromise outbreak surveillance for arboviral burden estimates. Syndromic diagnosis based on the 2009 WHO dengue classification [22] remain the frontline approach, but while sensitive (87% to 95%), it lacks specificity (6% to 20%) [23–25], and cannot reliably distinguish dengue from other febrile illnesses. Such diagnostic uncertainty can lead to inappropriate patient management, and distort surveillance data, particularly during large outbreaks [2,26,27].

Therefore, there is an urgent need for sensitive, specific, and deployable molecular tools. Despite concerns about feasibility, molecular diagnostics, especially real-time RT-PCR, offer superior sensitivity and pathogen specificity, particularly during the early acute phase. Furthermore, multiplex PCR platforms capable of simultaneously detecting multiple arboviruses can optimize diagnosis in regions where several viruses co-circulate [28–30]. Although multiplex-PCRs have been evaluated in several studies, even if their samples are collected in the Amazon, most assessments are done in university or reference laboratories [31–33]. Their implementation and performance in decentralized, resource-limited setting has rarely been assessed. While many evaluations of RDTs and ELISAs have compared their performance under controlled or reference laboratory conditions [34–38], several multi-country studies have shown that RDT accuracy remains variable and often suboptimal even in national reference laboratories [39,40], highlighting the need for real-world evidence.

This study addresses these gaps by comparing the performance of several diagnostic approaches for dengue during a large outbreak in Yurimaguas, in the Peruvian Amazon. We evaluated clinical assessment, two commercially available RDTs, and an NS1/IgM ELISA, alongside three molecular methods: a newly developed and validated multiplex real-time RT-PCR detecting DENV, ZIKV, YFV and CHIKV (ZYDC-PCR), a previously established triplex RT-PCR targeting DENV, ZIKV and CHIKV (ZDC-PCR); and a reference serotype specific dengue RT-PCR. We also assessed the diagnostic performance of the ZYDC-PCR in a central laboratory in Lima and a regional hospital in the Peruvian Amazon to take into account the critical need for the decentralization of molecular tools in arboviral surveillance. Our primary objective was to determine the diagnostic accuracy of these different approaches for dengue detection, while our secondary objective was to evaluate the inter-laboratory reproducibility of the ZYDC-PCR assay across distinct laboratory settings.

## 2. Methods

### 2.1. Ethics statement

This study was conducted in accordance with the principles of the Declaration of Helsinki and of the International Conference Harmonization (ICH) guidelines, plus adhering to local laws and regulations. All procedures used in the relevant clinical and laboratory studies comply with national and European Union legislation regarding research on human beings.

The study protocol, the informed consent form and all key documents of the study were approved by the Ethics Committee of the Universidad Peruana Cayetano Heredia (UPCH) (Protocol Nº 209778, approval date: 16^th^ of December 2022), the Institutional Review Board of the Institute of Tropical Medicine of Antwerp (Protocol Nº ITG 1661/23, approval date: 23^rd^ of March 2023) and the Ethics Committee of the Antwerp University Hospital (Project ID 5484, approval date: 12^th^ of June 2023) for its evaluation and approval, prior to the enrolment of patients. Written informed consent was obtained from adults, and for minors, written assent from minors and written consent from parent/guardian.

### 2.2. Study design

In this prospective study, 271 febrile patients were included in the KUNASA (Keys to understand UNdifferentiated Acute fever Sickness in the Peruvian Amazon) study at the Hospital Santa Gema (HSG) in Yurimaguas, Peru from end of September 2023 until mid-June of 2024. The HSG constitutes a hospital with level II-1 and a health area of approximately 80,000 inhabitants. Patients presenting with acute febrile illness (AFI) were included in the study. Eligibility criteria were temperature ≥ 38 °C lasting for 7 days or less, aged between 5–65 years old, regardless of gender and ethnicity, and the presence of at least one of the following symptoms: arthralgia, myalgia, headache, rash, nausea/vomiting. Exclusion criteria were febrile patients admitted in the hospital with acute respiratory infection (ARI), diarrhea and urinary tract infection (UTI), an identifiable disease (malaria or leptospirosis), with a fever episode of more than seven days, or a record of hospitalization in the preceding 2 weeks. Only individuals willing and able to provide written informed consent (including assent for minors older than 5 years) were included. Participants were registered according to HSG procedures and received routine medical care.

In Yurimaguas (Hospital Santa Gema), malaria was excluded by tick blood smear microscopy, leptospirosis was evaluated using an IgM/IgG rapid diagnostic test (RDT) (JusChek, Ref# ILEP-402).

Information from the patient was recorded in the official epidemiological form for the surveillance of dengue, Zika, chikungunya, yellow fever and other arboviruses provided by the Ministry of Health. This was done by the treating physician as part of routine care and includes: patient identification, socio-demographic information, clinical and epidemiological data. The last sections include lab assays ordered by the clinician and the disease classification based on the clinical and laboratory results. Although laboratory results are later incorporated into the form, they do not influence the initial clinical assessment used in this study. The form recorded hospitalization status but not the evaluation setting or admission rationale. Per national guidelines, warning signs typically warrant hospitalization, though final decisions are made by the treating physician.

Clinicians performed the WHO 2009 dengue classification during routine clinical evaluation before any laboratory assays had been conducted, ensuring that clinical classification was performed independently of laboratory data. Laboratory personnel conducting diagnostic assays were aware that samples corresponded to suspected dengue cases but were not provided with any individual clinical data.

Venous blood samples collected from the participants were processed at the lab facilities of the HSG, stored at -80°C and shipped to UPCH Lima for long-term storage.

### 2.3. Laboratory confirmation assays

#### 2.3.1. Rapid Diagnostic Tests (RDT) for DENV.

Two commercial RDTs were performed on the serum samples according to manufacturer’s instructions. A first screening for DENV was performed at Santa Gema Hospital using the Standard Diagnostics (SD) BIOLINE Dengue Duo Non-structural (NS)-1 Ag/IgM/IgG combo test (Abbott). This test was performed on-site immediately after patient admission. Another RDT for DENV using the OnSite Duo Dengue Ag-IgG/IgM Rapid Test (CTK Biotech) was done by the hospital. Following manufacturer’s instructions, a faint or strong line in the NS1 or IgM antibody tests was considered positive. IgG antibody results were not included in the analysis, as they are not used to confirm acute infection in current clinical practice.

#### 2.3.2. ELISA for DENV.

The Dengue IgM Capture ELISA (Ref.: M1018, Vircell) and Platelia Dengue NS1 Ag ELISA (Ref.: #72830, Bio-Rad) were performed according to manufacturer’s instructions at the *Laboratorio Referencial de Salud Pública del Alto Amazonas*. Both assays were processed and interpreted independently by the laboratory’s technical staff as part of routine diagnostic procedures. This study did not interfere with or influence the laboratory’s workflow. Access to results was obtained retrospectively through the patient’s epidemiological records. Results were recorded as positive, negative or indeterminate.

#### 2.3.3. Molecular assays.

**2.3.3.1. Nucleic acid extraction:** The nucleic acid extraction was performed with the QIAamp Viral RNA Mini kit (Qiagen, Germany), following the manufacturer’s instructions. The elution was done in two steps with 40µl of elution buffer and 5 min of incubation. Extracted RNA was stored at -80°C until use.

**2.3.3.2. Reverse Transcription Polymerase Chain Reaction (RT-PCR):** Three different RT-PCR assays were used for the molecular diagnosis of acute febrile illness in this study: i) Serotype-specific dengue PCR: as the reference test for dengue diagnosis, allowing identification of individual DENV serotypes [41], ii) Multiplex ZDC-PCR: an in-house triplex RT-PCR assay targeting ZIKV, DENV, and CHIKV, previously implemented at the Instituto de Medicina Tropical Alexander von Humboldt [42–44], and iii) ZYDC-PCR: a novel multiplex assay for ZIKV, YFV, DENV, and CHIKV, previously validated at the Institute of Tropical Medicine in Antwerp (manuscript accepted).

All RT-PCRs were performed using the iTaq Universal Probes One-Step Kit (Bio-Rad, USA) on a Bio-Rad Opus instrument. The final reaction volume was 25µL consisting of 5µL RNA, 12.5µL of iTaq Universal Buffer, 0.5µL of iScript RT, target-specific concentrations of primers and probes (see S1 and S2 Table), and nuclease-free water. The cycling conditions were: Step 1 at 50°C for 600 seconds (reverse transcription), step 2 at 95°C for 300 seconds, step 3 at 95°C for 10 seconds, and step 4 at 60°C for 30 seconds. Steps 3 and 4 were repeated 50 times. Baseline subtracted curve fit and single threshold were used as set by the Bio-Rad Opus data analysis program. A cut-off Cq value of 39 was defined to all assays, values above this threshold were classified as indeterminate. For the diagnostic performance calculations, samples with indeterminate PCR results (Cq > 39) were omitted from the analysis.

### 2.4. Comparison of the ZYDC-PCR with two other RT-PCR assays

To assess diagnostic performance, the ZYDC-PCR was compared with two additional molecular assays: the serotype-specific dengue PCR [41], used as the reference method for all performance calculations (i.e., sensitivity, specificity), and the in-house multiplex ZDC-PCR assay for ZIKV, DENV and CHIKV [42–44]. All three assays were implemented at the UPCH in Lima. The PCRs were run in parallel using the same set of RNA samples (n = 271) and distributed across multiple plates. To ensure consistency and comparability of results, each plate included positive and negative controls. All reactions were done in a Bio-Rad CFX Opus real-time PCR machine.

### 2.5. Inter-Laboratory verification of ZYDC-PCR results

An inter-laboratory verification was conducted to assess reproducibility using a subset of 131 serum samples from the total of 271 samples included in the study. These 131 samples correspond to patients enrolled after the implementation of the ZYDC-PCR at HSG in Yurimaguas. The first 89 collected samples had been shipped to Lima prior to the implementation of the assay in HSG and therefore could not be included for this verification.

For the inter-laboratory comparison, RNA extraction and ZYDC-PCR were initially performed at HSG. The same 131 samples were subsequently shipped to UPCH in Lima, where RNA extraction and ZYDC-PCR were performed by a different operator.

Quality control measures included internal extraction controls, positive and negative PCR controls, and technical replicates. As internal control, serum samples were spiked with the culture supernatant of phocine distemper virus (PDV), an RNA virus. To monitor extraction efficiency and detect potential inhibition, a PDV RT-PCR was performed at UPCH on around 30% (n = 36) of the RNA extracts generated in Yurimaguas.

### 2.6. Statistical analysis

All statistical analyses were conducted using R (version 4.4.3) https://www.r-project.org/. Continuous variables were summarized using medians and interquartile ranges (IQRs) or 95% confidence intervals (CIs) and categorical variables as counts and proportions. The Wilcoxon rank-sum test for continuous variables and the chi-square (χ²) test or Fisher’s exact test for categorical variables were used to assess differences between groups.

Logistic regression models were used to evaluate the association between dengue positivity and clinical symptoms (odds ratios (ORs) and 95% CIs) and between dengue serotype and clinical severity.

Multiple linear regression analysis with Cq value as the dependent variable and DENV serotype as the primary predictor was performed. The model included days after symptom onset (DASO) as a covariate to adjust for the effect of time on viral load. Statistical significance was set at *p* < 0.05.

Bland-Altman analysis was used to assess the difference in Cq values, comparing the two multiplex-PCRs against the reference, and the results of the ZYDC-PCR performed in Yurimaguas and Lima. The mean difference (bias) and 95% limits of agreement (LoA) were calculated.

Sensitivity, specificity, with their corresponding 95% CIs, were used to evaluate the diagnostic performance of the RDTs, with the ZYDC RT-PCR as the reference standard. The diagnostic metrics were calculated for individual RDT components (NS1 and IgM) and their combinations (NS1 + IgM) across different brands and formats. To visualize the comparative accuracy of these tests, a forest plot was made. Some samples did not have results from the RDT or ELISA assay due to routine constraints. For each assay, diagnostic performance metrics were calculated using only the subset of samples with available results, and no imputation of missing data was performed.

To further assess the added value of molecular diagnostics over clinical or serological approaches, a logistic regression and ROC analysis were made. A series of binary logistic regression models were fitted using the glm() function in R with a binomial family and logit link function to predict dengue positivity, defined by a positive result on the reference serotype-specific dengue RT-PCR. Models were performed comparing the predicted performance of clinical symptoms alone, against clinical symptoms with one added laboratory test. No model was used for the ELISA IgM due to low sample number. Five logistic models were compared: i) model 1 with the clinical symptoms that were significant in our analysis (rash, arthralgia in the hands and abdominal pain) as predictor, ii) model 2 with the addition of the ZYDC-PCR as predictor, iii) model 3 with the addition of the NS1 results of the SD Bioline RDT as predictor, iv) model 4 with the addition of the NS1 results of CTK RDT as predictor and v) model 5 with the addition of the results of the NS1-ELISA as predictor. Predicted probabilities were calculated with the predict function with type = “response”. The predictive performance of each model was evaluated using receiver operating characteristic (ROC) curves and area under the curve (AUC) generated with the pROC package. Pairwise comparison of AUCs between models were done using DeLong’s test for two correlated ROC curves (roc.test()), with a statistical significance set at p < 0.05.

## 3. Results

### 3.1. Clinical diagnosis and correlation with molecular confirmation

A total of 271 patients presenting with acute febrile illness were included in this study. The reference serotype-specific PCR confirmed DENV infection in 88 (32.6%), was negative in 182, while one patient had an indeterminate PCR result. The analyses were restricted to the 270 patients with a definitive reference PCR result. The median days after symptom onset (DASO) was 2 days (IQR: 1–4). Based on the WHO 2009 dengue guidelines, physicians classified each patient as dengue without warning signs (DwoWS)**,** dengue with warning signs (DwWS), or severe dengue [22].

To evaluate the diagnostic accuracy of clinical assessment, we compared WHO 2009 dengue classification against PCR-confirmed dengue status. Among the 261 patients with a recorded clinical classification, 210 (77.8%) were classified as DwoWS and 51 (18.9%) as DwWS. The risk of a suspected dengue case to be classified as a confirmed dengue case by PCR was significantly higher among patients classified as DwWS (73.33%, 95% CI: 66.8–79.2%) compared to those classified as DwoWS (45.1%, 95% CI: 31.1–59.7%; *p* < 0.001). This corresponds to a risk ratio of 1.63 (95% CI: 1.19–2.23) and an odds ratio (OR) of 3.35 (95% CI: 1.78–6.29), indicating that patients classified as DwWS had a 63% higher risk and over threefold higher odds of being PCR-positive than those with DwoWS. The association remained significant after adjusting for age and gender in a multivariable logistic regression (adjusted OR = 3.37, 95% CI: 1.79–6.33, *p* < 0.001). This indicates that the DwWS category is more reliable in capturing clinically apparent dengue cases, while the DwoWS group includes a higher proportion of patients with dengue-like symptoms caused by other etiologies.

In our study, 54 patients were hospitalized, of whom 32 (59.3%) were confirmed DENV-positive by PCR. Among the 37 hospitalized patients classified as DwWS, 25 were PCR-positive, indicating that approximately one-third of DwWS hospitalizations were not caused by dengue.

Logistic regression analysis was performed to evaluate the association between individual symptoms and PCR-confirmed dengue diagnosis. Dengue positivity was significantly associated with rash (OR: 2.31, 95% CI: 1.09–4.89, *p* = 0.028), arthralgia in hands (OR: 2.12, 95% CI: 1.19–3.89, *p* = 0.013) and abdominal pain (OR: 2.34, 95% CI: 1.15–4.79, *p* = 0.019). Symptoms such as myalgia, headache, retro-ocular pain, lumbar pain, conjunctivitis, vomiting and nausea, were present among DENV positive and DENV negative individuals, and the logistic regression analysis did not show significant association. For symptoms with counts less than 5 among the patients (mucosal bleeding, reduced urine output, hematocrit, hypothermia, liver enlargement, jaundice, altered mental status), the Chi-squared test was not performed due to violation of test assumptions (Table 1).

**Table 1:** Demographic and clinical description of the 271 acute undifferentiated fever patients enrolled in the study.

Demographics	PCR DENV positive n = 88 (32.6%)	PCR negative n = 182 (67.4%)	Total n = 270	*p-value* ^*$*^
Age (years)				0.112
5-19	29 (33.0%)	66 (36.3%)	95 (35.2%)	
20-44	49 (55.7%)	81 (44.5%)	130 (48.1%)	
45-59	8 (9.1%)	33 (18.1%)	41 (15.2%)	
> 60	2 (2.3%)	2 (1.1%)	4 (1.5%)	
Gender				0.038
Female	60 (68.2%)	100 (54.9%)	160 (59.3%)	
Male	28 (31.8%)	82 (45.1%)	110 (40.7%)	
WHO clinical classification				<0.001
woWS	56 (63.6%)	154 (84.6%)	210 (77.8%)	
wWS	28 (31.8%)	23 (12.6%)	51 (18.9%)	
Severe dengue	0 (0.0%)	0 (0.0%)	0 (0.0%)	
Unclassified	4 (4.5%)	5 (2.7%)	9 (3.3%)	
Median days after symptom onset (DASO)	2 (Q1-Q3: 1–4)	2 (Q1-Q3: 1–4)	2 (Q1-Q3: 1–4)	
Hospitalized	32 (59.3%)	22 (40.7%)	54 (20.0%)	<0.001
Symptoms*				
Fever	88 (32.6%)	182 (67.4%)	271 (100%)	
Arthralgia hand	69 (78.4%)	115 (63.2%)	184 (68.1%)	0.012
Arthralgia foot	60 (68.2%)	115 (63.2%)	175 (64.8%)	0.420
Myalgia	65 (73.9%)	123 (67.6%)	188 (69.6%)	0.293
Headache	65 (73.9%)	126 (69.2%)	191 (70.7%)	0.433
Eye pain	45 (51.1%)	85 (46.7%)	130 (48.1%)	0.494
Nausea	35 (39.8%)	74 (40.7%)	109 (40.4%)	0.889
Lumbar pain	32 (36.4%)	48 (26.4%)	80 (29.6%)	0.092
Abdominal pain	18 (20.5%)	18 (9.9%)	36 (13.3%)	0.017
Rash	16 (18.2%)	16 (8.8%)	32 (11.9%)	0.025
Vomiting	8 (9.1%)	10 (5.5%)	18 (6.7%)	0.267
Conjunctivitis	6 (6.8%)	10 (5.5%)	16 (5.9%)	0.666

* Percentages are calculated based on the total number of DENV positive or negative samples. ^$^ Fisher’s exact test; Pearson’s Chi-squared test.

DENV2 was the most prevalent serotype (49/88, 55.7%), followed by DENV3 (24/88, 27.3%) and DENV1 (15/88, 17.0%). Although the proportion of DENV cases classified as DwWS varied by serotype, these differences were not statistically significant (see S3 Table). In a multivariable logistic regression evaluating serotype as a predictor of DwWS (adjusted for age, sex and DASO), no serotype showed an independent association with the presence of warning signs (*p* > 0.05; S4 Table).

The median Cq value of the reference serotype-specific PCR among confirmed dengue cases was 21.6 (IQR: 18.5–31.1). Cq values increased with DASO (R = 0.47, *p* < 0.001) reflecting the expected decline in viral load over course of illness. After adjusting for DASO In the linear regression model, each additional day after symptom onset, was associated with 2.15 cycle increase in Cq value (*p* < 0.001). Compared with DENV1, DENV2 infections showed an average increase of 4.29 Cq cycles (*p* = 0.031), and DENV3 infections with a 5.85 cycle higher Cq (*p* = 0.009), consistent with lower viral loads in these serotypes (S5 Table).

### 3.2. Comparison of PCR results with serological commercial assays for acute DENV suspected cases

**Fig 1:**
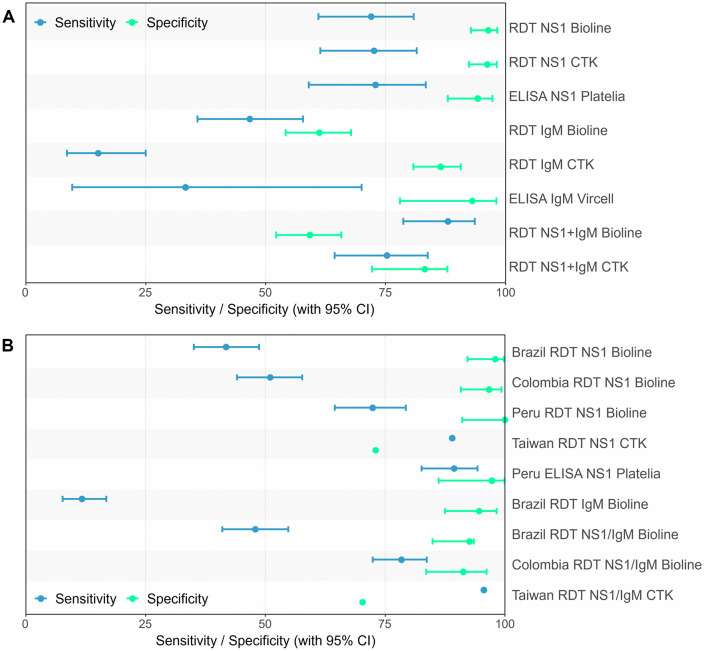
Performance comparison of different rapid diagnostic tests and ELISA. **A)** Forest plot of the performance of the RDTs and ELISAs for NS1 and IgM, compared with the ZYDC-PCR based on this study. **B)** Forest plot of the performance of RDTs and ELISA for NS1 and IgM from studies performed in different countries based on the review from Haider et al. [45] and from Pal et al for the Peru ELISA NS1 Platelia [37]. Sensitivity and specificity estimates are plotted on the x-axis (0–100%), with corresponding 95% confidence intervals. Tests are displayed along the y-axis.

The SD Bioline RDT was performed on 271 samples, the CTK RDT on 258 samples, the NS1 ELISA on 152 samples and the IgM ELISA on 35. Due to the one sample with the indeterminate result in the ZYDC-PCR, that was omitted, the Bioline RDT analysis was performed on 270 samples, the CTK RDT on 257 samples, the NS1 ELISA on 152 samples and the IgM ELISA on 35. The performance characteristics for NS1 were highly similar between the two RDTs. Bioline had a sensitivity of 72.0% (95% CI: 61.0-80.9) and a specificity of 96.4% (95% CI: 92.8-98.3), whereas the CTK showed a sensitivity of 72.6% (95% CI: 61.4-81.5) and a specificity of 96.2% (95% CI: 92.4-98.2) (see S6 Table and Fig 1).

Cohen’s kappa value showed a substantial agreement with the reference test, with 0.726 for Bioline and 0.727 for CTK. In the subset of 149 samples with results for both RDTs and the NS1 ELISA, the NS1 component of the RDTs showed similar sensitivity (72.2%, 95% CI: 65.7-88.3) and high specificity (94.1%, 95% CI: 87.6-97.2) as the ELISA (S7 Table). Cohen’s kappa values indicated substantial agreement across all three NS1 tests (Bioline and CTK κ = 0.749, NS1 ELISA κ = 0.600).

A significant difference in Cq values was observed between true positive and false negative samples for the NS1 RDTs and ELISA; samples with lower Cq values (i.e., higher viral loads) were more likely to be correctly identified by an NS1-based test (S1 Fig).

In contrast, IgM performance for both RDTs was poor. Bioline sensitivity was 46.7% (95% CI: 35.8-57.8) and specificity 61.0% (95% CI: 54.0-67.6), whereas for the CTK sensitivity was 15.1% (95% CI: 8.6-25.0) and specificity 86.4% (95% CI: 80.7-90.6). Both had minimal agreement with the reference PCR (Cohen’s kappa: 0.067 for Bioline and 0.017 for CTK) (S6 Table). When NS1 and IgM results were combined, sensitivity improved to 88.0% (95% CI: 78.8-93.6) for Bioline and to 75.3% (95% CI: 64.4-83.8) for CTK, but the specificity decreased to 59.0% (95% CI: 52.0-65.6) and 83.2% (95% CI: 77.1-87.9), respectively. Cohen’s kappa value showed only fair agreement for the Bioline RDT (κ = 0.364) and moderate agreement for the CTK RDT (κ = 0.555).

The IgM ELISA was performed on a smaller subset of 35 samples and demonstrated low sensitivity (33.3%, 95% CI: 9.7-70.0) but high specificity (93.1%, 95% CI: 78.0-98.1), with a fair agreement to PCR (Cohen’s kappa, κ = 0.305).

When stratifying NS1 and IgM test performance by dengue serotype, some variability in sensitivity was observed—particularly lower values for DENV3 across both RDTs and the ELISA. However, interpretation is limited by the small sample size per serotype group (15 for DENV1, 49 for DENV2, 24 for DENV3) (S8 Table).

### 3.3. Performance comparison of the ZYDC-PCR and the ZDC-PCR with the reference PCR

Among the 271 febrile patients included in the study, three samples were omitted from the analysis due to indeterminate results in their Cq values. This included one result from the reference serotype-specific PCR, one from the ZYDC-PCR and one from the ZDC-PCR.

Of the remaining 268 samples, 86 (32.1%) were confirmed as dengue-positive by the serotype-specific dengue PCR, which was used as the reference of comparison. The ZYDC-PCR detected 74 out of the 86 cases identified as positive by the reference PCR (sensitivity 86.0%, 95% CI: 77.2-91.8), while the ZDC-PCR identified 65 of these samples as positive (sensitivity 75.6%, 95% CI: 65.5-83.4). Clinical specificity was 100% for both assays. Kappa statistics indicated strong agreement with the reference serotype-specific dengue PCR, with values of 0.893 for the ZYDC-PCR and 0.808 for the ZDC-PCR.

None of the samples tested positive for CHIKV, ZIKV or YFV with the ZYDC-PCR or ZDC-PCR.

The median Cq value of the 12 false negative samples in the ZYDC-PCR, was 35.5 (IQR: 34.4 to 38.2), as determined by the reference serotype-specific dengue PCR. When stratified by serotype, the median Cq values were 33.8 (n = 3, IQR: 33.4 to 34.6) for DENV1, 35.7 (n = 4, IQR: 34.6 to 36.6) for DENV2 and 37.3 (n = 5, IQR: 37.5 to38.2) for DENV3 (Table 2).

**Table 2:** Performance comparison of the ZYDC-PCR and ZDC-PCR with the reference PCR.

Total n=(268)	Serotype-specific PCR	ZYDC-multiplex	ZDC-multiplex	Median Cq value of samples not detected with ZYDC-multiplex
DENV neg	182 (67.9%)	194 (72.4%)	203 (75.7%)	
DENV pos	86 (32.1%)	74 (27.6%)	65 (24.3%)	35.5 (IQR: 34.4-38.2)
DENV1	15 (17.4%)	12 (16.2%)	11 (16.9%)	33.8 (IQR: 33.4-34.6)
DENV2	48 (55.8%)	44 (59.5%)	39 (60.0%)	35.7 (IQR: 34.6-36.6)
DENV3	23 (26.7%)	18 (24.3%)	15 (23.1%)	37.3 (IQR: 37.5-38.2)
Sensitivity	Reference	86.0% (CI: 77.2%-91.8%)	75.6% (CI: 65.5-83.4%)	
Specificity	Reference	100% (CI: 97.9-100%)	100% (CI: 97.9-100%)	
kappa	Reference	0.893	0.808	

The reference PCR (serotype-specific dengue PCR) and the ZYDC-multiplex and ZDC-multiplex PCR were performed at the central laboratory at UPCH in Lima.

**Fig 2:**
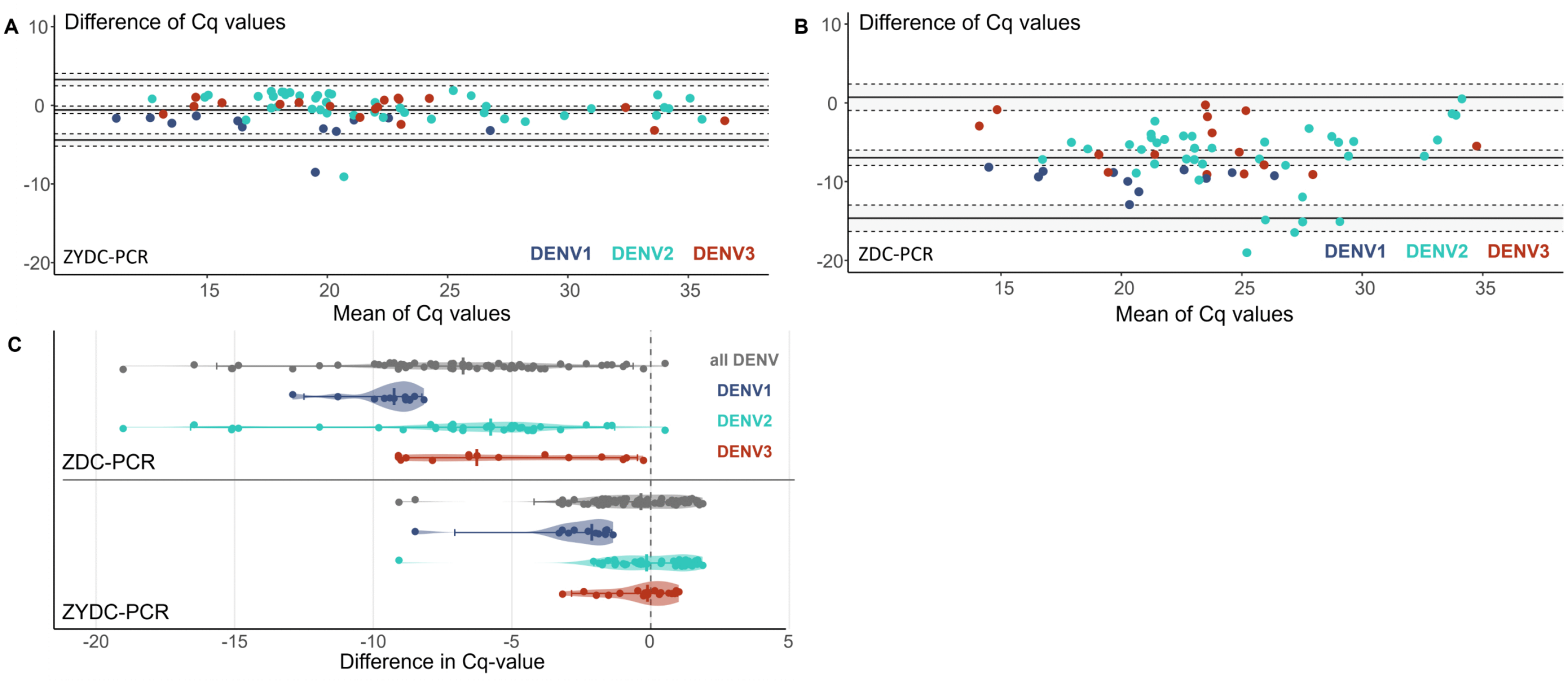
Comparison of Cq values obtained with the ZYDC-multiplex PCR and ZDC-multiplex PCR relative to the reference PCR. For each sample, Cq values obtained with the serotype-specific reference dengue PCR were compared to those obtained with the multiplex assays (ZYDC-PCR and ZDC-PCR). Bland-Altman plot showing the difference inCq values between the reference PCR and the ZYDC-multiplex PCR **A)** and the ZDC-multiplex PCR **B)** plotted against the mean Cq value. **C)** Violin plots showing the distribution of Cq value differences (multiplex PCR minus reference PCR) for the ZYDC-PCR and ZDC-PCR, stratified by dengue serotype (DENV1, DENV2, DENV3 and all DENV combined)**.**

The Bland-Altman plot (Fig 2A) comparing the Cq values of the ZYDC-PCR to the reference serotype-specific dengue PCR showed no systematic bias and a good agreement, with the mean difference close to zero (-0.6) and most data points within the 95% limits of agreement. In contrast, the Bland-Altman plot (Fig 2B) for the ZDC-PCR showed a systematic bias, with the ZDC-PCR consistently yielding higher Cq values than the reference PCR. The mean difference in the Cq value for the ZDC-PCR was 6.9 cycles lower than the difference of the ZYDC-PCR with the reference serotype-specific dengue PCR.

As can be seen in the violin plot in Fig 2C, the median Cq difference between the reference PCR and the ZYDC-PCR was -0.3 (IQR: -1.6 to 0.9). The difference in Cq-values was highest for DENV1 with -2.1 (IQR: -3.0 to -1.6), while smaller differences were found for DENV2 (-0.1; IQR: -1.0 to 1.2) and DENV3 (-0.1; IQR: -0.9 to 0.6). Comparing the ZDC-PCR and the reference PCR, the median Cq difference was -6.8 (IQR: -8.9 to 4.6). This difference was -9.2 (IQR: -9.8 to -8.8) for DENV1, -5.8 (IQR: -7.8 to -4.5) for DENV2 and -6.3 (IQR: -8.3 to -2.4) for DENV3 (Fig 2C).

### 3.4. Inter-laboratory verification of ZYDC-PCR

Out of the 131 samples tested in Yurimaguas, five samples were omitted in the analysis, due to their Cq value of over the threshold 39 for indeterminate results, this included one sample tested with the reference PCR in Lima and four with the ZYDC-PCR in Yurimaguas. Of the 126 samples included in this analysis, 58 tested positive for DENV based on the reference serotype-specific dengue PCR performed in Lima. Among these, 50 were also detected by the ZYDC-PCR in Lima, and 52 by ZYDC-PCR in Yurimaguas.

Two different dilutions of PDV were used, with one resulting in a mean Cq value of 26.8 (SD: 0.41; n = 8) and the other with a mean Cq value of 34.0 (SD 2.5; n = 28).

**Fig 3:**
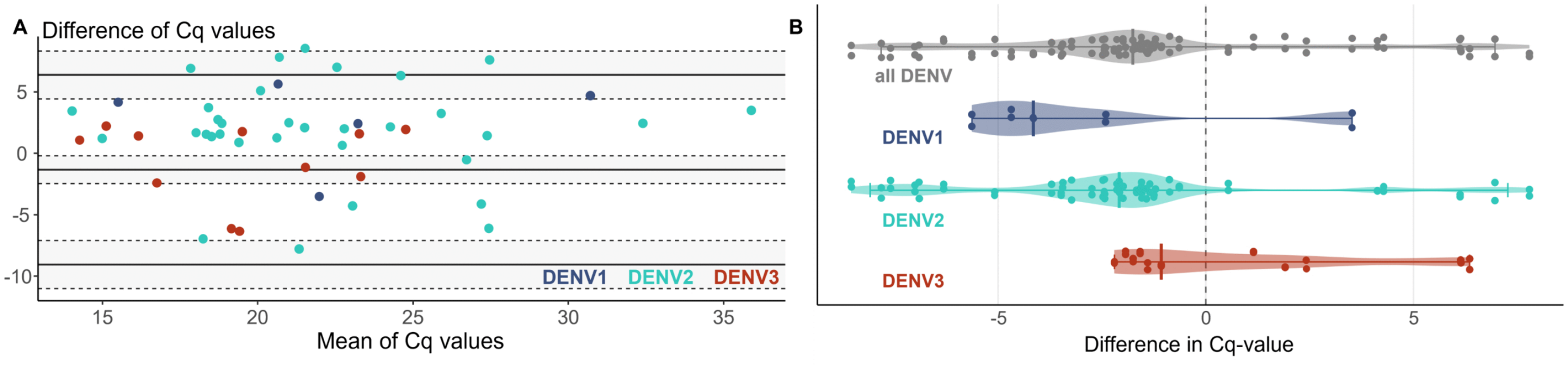
Comparison of the Cq value results of the ZYDC-PCR performed in Yurimaguas and Lima. Cq values obtained with the ZYDC-PCR in Yurimaguas and Lima were compared using **A)** a Bland-Altman plot and **B)** a Violin plot, stratified by dengue serotype**.**

The Bland-Altman plot showed poor agreement in the Cq values obtained from the ZYDC-PCR conducted in Yurimaguas versus Lima (Fig 3A). While the mean difference (bias) was relatively small at -1.4, suggesting minimal systematic deviation between sites, the wide limits of agreement (LOA: -9.1 to 6.4), indicated substantial variability in individual measurements, reflecting inconsistent performance of the assay between the two laboratories. A comparable dispersion was observed in a subset of samples, where the RNA extracted in Yurimaguas was run with the serotype-specific dengue PCR in Lima (see S2 Fig). The mean difference (bias) was similar to the comparison of the ZYDC-PCR with -1.2 and wide limits of agreement (LOA: -6.6 to 4.3). The differences were statistically significant (Wilcoxon rank test p-value <0.001), even after removal of nine outliers. There was a systematic bias with lower Cq values in Lima than in Yurimaguas, with a median difference of -1.8 cycles (IQR: -3.4 to -0.6) across all DENV-positive samples. The differences were -4.1 cycles (IQR: -4.7 to -2.4) for DENV1, -2.1 (IQR: -3.5 to -1.2) for DENV2, and -1.1 (IQR: -1.7 to 2.2.) for DENV3. However, the large IQR indicated marked variability in the measurement between laboratories, as illustrated in the violin plot in Fig 3B. The sensitivity of the ZYDC-PCR was similar across laboratories, with 89.7% (95% CI: 79.2 to 95.2; Cohen’s kappa, κ = 0.903) for Yurimaguas and 86.2% (95% CI: 75.1 to 92.8); Cohen’s kappa, κ = 0.871) for Lima, when compared to the reference PCR (Table 3).

**Table 3:** Inter-laboratory comparison.

Total (n = 126)	Serotype- specific PCR	ZYDC-PCR in Yurimaguas	ZYDC-PCR in Lima
DENV negatives	68 (54.0%)	74 (58.7%)	76 (60.0%)
DENV positives	58 (46.0%)	52 (41.3%)	50 (39.7%)
DENV1*	8 (13.8%)	5 (9.6%)	5 (10.0%)
DENV2*	35 (60.3%)	35 (67.3%)	33 (66.0%)
DENV3 *	15 (25.9%)	12 (23.1%)	12 (24.0%)
Diagnostic Performance			
Sensitivity	Ref.	89.7% (CI: 79.2-95.2%)	86.2% (CI: 75.1-92.8%)
Specificity	Ref.	100% (CI: 94.7-100.0%)	100% (CI: 94.7-100.0%)
kappa	Ref.	0.903	0.871

Comparison of the ZYDC-PCR for 126 samples tested in Yurimaguas and in Lima, using the serotype-specific dengue PCR as the reference standard. * Percentages are calculated based on the total number of DENV positive samples. Kappa statistics indicates agreement between assays.

### 3.5. Samples with discordant results across PCR assays

Three samples that tested positive by both the reference serotype-specific dengue PCR (Cq values: 35.54, 34.75, 36.22) and the ZYDC-PCR in Lima (37.50, 38.45, 41.38), were not detected when tested in Yurimaguas by the same ZYDC-PCR assay (Table 4).

**Table 4:** Comparison of Cq values across diagnostic PCR assays for discrepant samples.

Sample	DENV serotype	Serospecific DENV- PCR	ZYDC-PCR Yurimaguas	ZYDC-PCR Lima
1	DENV1	34.40	ND	ND
2	DENV3	40.30	ND	ND
3	DENV3	38.31	ND	ND
4	DENV1	32.33	ND	ND
5	DENV3	37.54	ND	ND
6	DENV3	35.54	ND	37.50
7	DENV1	34.75	ND	38.45
8	DENV3	36.22	ND	41.38
9	DENV2	38.85	41.96	ND
10	DENV2	33.11	43.26	33.54
11	DENV3	32.01	39.41	35.19
12	DENV2	ND	43.18	ND

ND = not detected.

Additionally, five samples that tested positive in the reference serotype-specific dengue PCR (Cq values: 34.40, 40.30, 38.31, 32.33, 37.54) were not detected by the ZYDC-PCR in either Lima or Yurimaguas. One sample with a Cq value of 38.85 in the reference serotype-specific dengue PCR and 41.96 with the ZYDC-PCR in Yurimaguas tested negative with the ZYDC-PCR in Lima. Another sample yielded a Cq value of 43.18 with the ZYDC-PCR in Yurimaguas, above the indeterminate threshold, but tested positive for DENV2 (Cq = 38.05) when retested using only the simplex-DENV2 reference PCR on the same RNA extracted in Yurimaguas. This confirms the importance to treat high Cq values (>39) as “indeterminate” rather than negative and to confirm such cases with a reference assay.

### 3.6. Comparison of the predictive performance of the different diagnostic approaches

**Fig 4:**
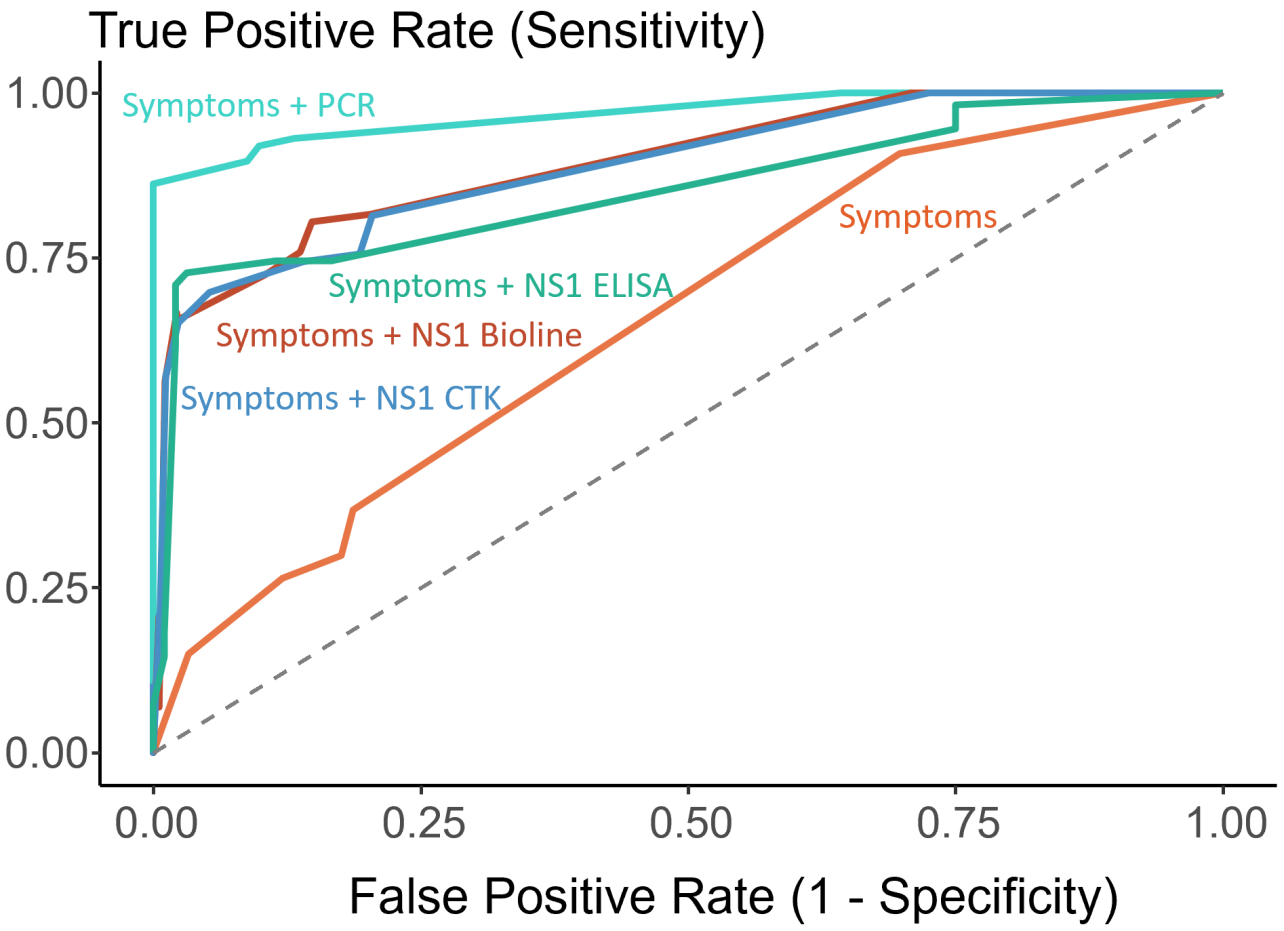
Receiver operating characteristic (ROC) curves comparing the predictive performance of logistic regression models for dengue diagnosis. The reference standard was the serotype specific dengue PCR. Model 1 included clinical symptoms only (rash, abdominal pain and hand arthralgia) (AUC = 0.65); model 2 added the ZYDC-PCR results (AUC = 0.97); model 3 included the SD Bioline NS1 results (AUC = 0.89); model 4 included the CTK NS1 results (AUC = 0.89); and model 5 the NS1 ELISA results (AUC = 0.85).

The logistic regression model based on only clinical symptoms (fever plus either rash, abdominal pain or hand arthralgia) had a limited discriminative performance with an AUC of 0.65 (95% CI: 0.59 to 0.72), indicating that a substantial proportion of patients would be misclassified in the absence of laboratory confirmation. The inclusion of any laboratory test significantly improved diagnostic accuracy (see Fig 4).

The largest improvement was seen when adding the ZYDC-PCR to the model (AUC of 0.97, 95% CI: 0.95 to 0.99). Models incorporating NS1 testing also showed an increase in performance, with AUC = 0.89 (95% CI: 0.85 to 0.93) for SD Bioline RDT, AUC = 0.89 (95% CI: 0.85 to 0.93) for CTK RDT and AUC = 0.85 (95% CI: 0.79 to 0.92) for the NS1-ELISA.

Statistical comparisons using DeLong’s test confirmed that all models incorporating laboratory diagnostics significantly outperformed the symptom-only model, with significant p-values: ZYDC-PCR model (*p* < 0.001), SD Bioline model (*p* < 0.001), CTK based model (*p* = 0.005) and NS1-ELISA based model (*p* = 0.011).

## 4. Discussion

Our study provides strong evidence that clinical diagnosis alone is insufficient for accurate dengue case confirmation. While the addition of NS1-based tests improved diagnostic performance from clinical symptoms alone, the most significant improvement was observed with the multiplex RT-PCR (ZYDC-PCR), which outperformed all other approaches, highlighting the value of molecular tools implementation in resource-limited settings.

An important finding of this study is the co-circulation of three dengue serotypes (DENV-1, DENV-2, DENV-3) in Yurimaguas during the 2024 outbreak. Historically, dengue epidemics in Peru have been dominated by a single serotype, with shifts occurring every few years [46–48]. The simultaneous detection of multiple serotypes suggests possible progression toward hyperendemic transmission in the Peruvian Amazon region [49], as have been reported for other countries [50]. This has important epidemiological implications as multi-serotype circulation increases the likelihood of secondary infections, which are associated with higher risk of severe disease [51,47,52–55] and might complicate vaccine impact assessments [56]. Strengthening serotype-specific surveillance is therefore essential to better understand transmission dynamics and inform public health responses.

No cases of CHIKV, ZIKV, or YFV were detected, possibly reflecting low prevalence or seasonal variation in transmission [57]. Although none of the other arboviruses were detected during this outbreak, their inclusion in multiplex panels is important due to the epidemic nature of ZIKV, CHIKV, and YFV and their overlapping clinical presentation with dengue. This has also been shown by recent reports of CHIKV and YFV cases in Peru [58,59].

High sensitivity while maintaining adequate specificity is essential for both clinical care and public health strategies. Dengue case management is largely symptomatic, following WHO and national guidelines, therefore, treatment should not be delayed while awaiting laboratory confirmation.

Nevertheless, earlier access to accurate diagnostics could help refine admission decisions, as one-third of DwWS admissions in our cohort were PCR-negative. Although some of these patients may still have required hospitalization for other causes of acute febrile illness, timely confirmation or exclusion of dengue would support more informed clinical decision-making and more efficient use of hospital resources.

Misclassification of non-dengue illnesses as dengue can delay appropriate treatments, e.g., nonsteroidal anti-inflammatory drugs (NSAIDs), which are standard treatment for CHIKV, but contraindicated in treatment in dengue [60]; and empirical antibiotics are often started when viral illnesses are mistaken for bacterial infections [2,61–63]. Leptospirosis, an important cause of acute fever in the Peruvian Amazon, requires prompt antibiotic therapy and false-positive dengue diagnoses can delay life-saving treatment [2]. False negatives, however, may delay clinical monitoring and increase the risk of severe dengue complications, particularly in patients with warning signs [10,64,65]. From surveillance perspective, underestimation of dengue due to false negatives can hinder outbreak detection and misguide resource allocation [10,60,61]. A notable example comes from Colombia, where three deaths initially reported as dengue were later confirmed as yellow fever during post-mortem investigations [66].

Although all enrolled patients were clinically classified as suspected dengue cases by the treating physician following the WHO 2009 criteria, only around one-third were PCR-confirmed, showing the low specificity of clinical assessment alone. This aligns with previous reports from Peru [4,67,68] and other endemic regions in Latin America [57,69–71], where in studies with similar inclusion criteria, substantial misclassification when relying solely on symptoms occurred.

Most of the confirmed dengue cases presented without warning signs, consistent with previous regional trends [72]. The low proportion of severe dengue (0.09% in Yurimaguas vs 0.27% nationally [73]) may reflect effective clinical management, host immunity or viral characteristics of the circulating DENV strain [22,51,74,75]. Notably, patients clinically classified as DwWS were more likely to be PCR-positive, suggesting greater clinical specificity in more symptomatic cases and supporting the utility of the 2009 WHO classification as a valuable clinical screening tool [76–78], fundamental to reduce the risk of complications [79,80]. However, some of the hospitalized patients with DwWS were PCR-negative for DENV, indicating that a substantial proportion may have been hospitalized precautionarily in the absence of rapid confirmatory testing. Earlier access to accurate diagnostics, either molecular or antigen-based, could help refine admission decisions, avoid unnecessary hospital stays, and allow clinicians to focus monitoring resources on patients at true risk of dengue complications.

The poor performance of IgM-based detection likely reflects the early timing of sample collection (median DASO = 2 days), before seroconversion [81,82]. No convalescent samples were available, as paired sampling is not recommended in endemic dengue areas. Secondary infections are common in endemic settings, and tend to have lower IgM responses, than primary infections [83,84]. These factors limit the usefulness of IgM assays for early clinical management and outbreak surveillance in settings such as Yurimaguas [21].

In our study, NS1-based tests showed moderate sensitivity (~70%) and high specificity (~95%), consistent with previous reports for RDTs [45,85–87] and NS1 ELISA [36,40,88]. The limitation in the sensitivity of NS1 detection could be attributed in part by variations in viral load across different disease phases. NS1 antigenemia peaks in the first few days of illness but rapidly declines thereafter [89], as evidenced also in our results with the significant difference in median Cq value of the false negative and true positive samples for the NS1 tests. NS1 antigen levels can vary by serotype and between primary and secondary infection, which may influence test performances [81]. Understanding and accounting for this variability will be essential for ensuring diagnostic test reliability across outbreak and non-outbreak settings.

The ease of use of RDTs supports continued deployment as a screening tool in low-resource settings, although negative results require cautious interpretation and more accurate confirmatory diagnostic tests. National diagnostic policies can vary significantly. In Peru, ELISA-based assays are accepted for confirmation, while RDTs are not [21,19]. In contrast, other dengue-endemic countries use RDTs for surveillance and reporting [90–92], and recently the WHO guidance on laboratory testing for DENV recommends their broader adoption [93]. Our findings show that NS1-based RDTs and NS1 ELISA assays have comparable diagnostic performance, suggesting that NS1 RDTs could offer a pragmatic alternative for remote or low-resource health centers where ELISA is operationally unfeasible. As ELISA requires trained personnel, and centralized infrastructure, typically found in Level II/III facilities (district hospitals or regional laboratories), its use may introduce considerable delays. In contrast, RDTs can be performed individually at the point of care, offering results within minutes and reduce the logistical challenges of transporting samples to reference laboratories. Given the high specificity of NS1 assays, a positive NS1 RDT result provides strong evidence of dengue infection. However, due to sensitivity limitations, RDT-negative cases should undergo PCR testing when available to avoid missed dengue cases or infections with other co-circulating arboviruses. Importantly, whenever PCR capacity exists, PCR should remain the test of choice for dengue confirmation.

Another limitation of current available NS1- or IgM-based diagnostic tests is that they are only detecting DENV, whereas PCR assays offer the advantage of multiplexing to simultaneously detect multiple pathogens. This capability is particularly valuable in regions where several arboviruses co-circulate. Our multiplex PCR targeted four pathogens (ZIKV, DENV, CHIKV, YFV) but given the diversity of other arboviruses circulating in Peru, inclusion of additional targets such as OROV, MAYV or VEEV would be valuable for differential diagnosis and surveillance. However, the number of pathogens that can be incorporated into a single multiplex-PCR is limited by the number of fluorescence detection channels available on real-time PCR machines (e.g., Biorad Opus supports five channels) and by the potential decrease in analytical sensibility when adding a higher number of primers and probes. Here we compared the performance of a newly implemented ZYDC-PCR with the ZDC-PCR already in use at the reference laboratory at UPCH in Lima and the serotype-specific dengue PCR as reference standard.

The ZYDC-PCR, which includes serotype-specific dengue primers, outperformed the ZDC-PCR already in use at UPCH in Lima, but had a lower sensitivity compared to the DENV serospecific reference PCR. This pattern is consistent with findings from other evaluations comparing dengue specific PCRs with multiplex assays, where multiplex PCRs often show reduced sensitivity with ranges of 65–68% [94], 73% [95], 91% [95] to 95% [95,96], and one study reporting 100% sensitivity [95]. In most cases, reduced sensitivity was attributed to lower viral load in the samples that fall below the detection threshold of the multiplex PCR, as was also the case with some of our samples. Although no ZIKV or CHIKV cases were detected during this outbreak, the capacity of the test to detect multiple arboviruses remains critical given their unpredictable emergence.

Despite the slight reduction in sensitivity for low-viremic samples of the multiplex ZYDC-PCR compared to the serotype-specific dengue PCR, the use of a multiplex PCR offers advantages over single-pathogen tests. YFV for example has increasing case numbers in Latin America in the recent years and has expanded outside of its sylvatic region in the Amazon [97]. In the last outbreak in Brazil, the virus circulated two years outside the Amazon before being detected, even in previously YFV-free regions of Southern Brazil [98]. A current outbreak of YFV in Latin America has spread to five countries (Bolivia, Brazil, Colombia, Ecuador and Peru) with 212 confirmed cases and 85 deaths [58].

The Americas are also currently experiencing an outbreak of CHIKV in 14 countries with 124,942 probable and confirmed cases and 110 deaths until August 2025. In Peru 84 cases were reported, mostly in the Amazon region, but only 16 were laboratory confirmed [59].

Timely access to reliable diagnostics is equally important, as even highly sensitive and specific tests offer limited value if results are delayed. In Peru, where local laboratory capacity is lacking, samples must be sent to the regional or national reference laboratories, a process for which the national guidelines allow up to 28 days [21]. Establishing molecular testing capacity within the hospital, or at least within the same city, would allow samples to be processed within 1–2 days, shortening the turnaround time. The successful operation of the ZYDC-PCR at Santa Gema Hospital shows that molecular testing can be performed outside reference laboratories, providing a proof-of-concept for decentralized diagnostics that could help mitigate long transport-related delays.

Our study showed variability across laboratories, despite the use of standardized protocols and reagents, and expertly trained staff. However, the diagnostic performance, in terms of sensitivity and specificity, was similar in Yurimaguas and in Lima for the ZYDC-PCR. One sample, classified as indetermined (Cq 43.18) in Yurimaguas by the ZYDC-PCR, was negative with the reference-PCR done in Lima (newly extracted RNA in Lima), but when the same RNA extracted in Yurimaguas was shipped and tested in Lima using the simplex DENV2 PCR only, the result was positive (Cq of 38.05). This shows the importance of processing samples at the place of collection to avoid potential nucleic acid degradation during storage, transportation and thaw-freeze cycles.

The systematic trend of lower Cq values in Lima compared to Yurimaguas suggests potential differences in sample handling, extraction efficiency, or PCR amplification conditions between the sites. One contributing factor may be differences in threshold settings of the two Opus machines. The automatically calculated thresholds in relative fluorescence units (RFU) in Lima (median 96.9 RFU, IQR 62.9 to 164.6 RFU) were consistently lower than in Yurimaguas (median 267.6 RFU, IQR 206.7 to 313.1 RFU).

The similar variability in Cq values in RNA extracted in Yurimaguas and re-tested in Lima with the serotype-specific dengue PCR, indicates that the issue was unlikely related to PCR amplification performance. Instead, it may reflect differences in RNA extraction or reagent stability. Although PCR kits were shipped on dry ice, the RNA extraction kit was transported at ambient temperature (manufacturer’s specifications: up to 25°C), and temperature fluctuations during transport or storage may have affected extraction efficiency. Future decentralized implementations should include regular inter-laboratory quality controls and careful management of supply chain conditions for extraction reagents.

Although reagents and extraction kits can be supplied to Amazonian regions through local distributors, prices are typically higher than in Lima due to cold-chain requirements and transport needs, particularly given the high ambient temperatures in Loreto. Nevertheless, performing PCR locally is likely more cost-efficient than shipping samples to Lima for centralized testing and provides substantially faster results, which is critical for clinical management and timely public health response. The successful implementation of ZYDC-PCR in Yurimaguas demonstrates technical feasibility, though broader adoption would require strengthened supply-chain logistics, infrastructure stability, and sustained personnel training.

This study has several limitations. Sample collection was done in one single site during one outbreak period, which limits the generalizability of our findings, especially with regards to outbreaks with circulation of several arboviruses. Our findings should be interpreted in the context of the 2024 outbreak in Yurimaguas, which was dominated by dengue and showed minimal co-circulation of other arboviruses. Diagnostic performance may differ in settings where multiple arboviruses circulate simultaneously. Seasonal fluctuations in arbovirus transmission, including periods when pathogens such as Oropouche virus, Mayaro virus, or CHIKV predominate, may also influence clinical presentation and alter the sensitivity or specificity of dengue diagnostic assays, particularly NS1- and IgM-based methods. Moreover, geographic heterogeneity in serotype circulation, transmission intensity, and background immunity could affect test performance in other Amazonian or coastal settings. These factors should be considered when extrapolating our results to broader epidemiological contexts.

Furthermore, since our samples were collected during the acute viremia, they were collected earlier than what is recommended to conduct IgM tests and the number of samples tested by IgM ELISA was small. These factors could have impacted their diagnostic performances. Although standardized protocols were used, inter-laboratory variability and environmental differences may have influenced lab-to-lab reproducibility.

Overall, our study indicates that clinical presentation alone is insufficient for reliable dengue diagnosis, particularly in settings where several AUFI etiologies co-circulate. NS1-based tests offer some diagnostic improvement, but their moderate sensitivity limits their utility for an early clinical management, especially for the high rate of false negatives. However, their simplicity might justify their use as a screening test in primary health care centers.

Implementing the ZYDC-PCR is technically feasible in a decentralized setting, although confirmation in larger scale studies is still needed. Scaling molecular diagnostics beyond pilot sites will require substantial systems-level strengthening, including infrastructure upgrades (power stability, equipment maintenance), reliable supply-chain and procurement mechanisms, functional laboratory information systems, standardized QA/QC and supervisory structures, and sustained investment in training and retention of qualified personnel. Beyond improving dengue detection, the multiplex PCR format offers the added advantage of enabling differential diagnostic of other co-circulating arboviruses, which is of the up most importance given recent yellow fever and chikungunya outbreaks in the region. Broader implementation of these molecular tools in regional hospitals could substantially reduce diagnostic delays, support more timely and appropriate patient management, and strengthen arboviral surveillance in endemic, resource-limited settings.

### Ethics approval and consent to participate

This study was conducted in accordance with the principles of the Declaration of Helsinki and of the International Conference Harmonization (ICH) guidelines, plus adhering to local laws and regulations. All procedures used in the relevant clinical and laboratory studies comply with national and European Union legislation regarding research on human beings.

The study protocol, the informed consent form and all key documents of the study were approved by the Ethics Committee of the Universidad Peruana Cayetano Heredia (Protocol Nº 209778, approval date: 16th of December 2022), the Institutional Review Board of the Institute of Tropical Medicine of Antwerp (Protocol Nº ITG 1661/23, approval date: 23rd of March 2023) and the Ethics Committee of the Antwerp University Hospital (Project ID 5484, approval date: 12th of June 2023) for its evaluation and approval, prior to the enrolment of patients. Written informed consent was obtained from adults, and for minors, written informed assent and caretaker consent were obtained.

## Supporting information

S1 Table Primers and probes of the ZYDC-PCR. (DOCX)

S2 Table Primers and probes of the ZDC-PCR and the reference PCR. (DOCX)

S3 Table Distribution of PCR-confirmed DENV infections with and without warning signs and their median Cq values. (DOCX)

S4 Table Multivariable logistic regression evaluating serotype as a predictor of dengue with warning signs (DwWS). (DOCX)

S5 Table Distribution of DENV positive samples with their median Cq value. (DOCX)

S6 Table Results for performance testing for the RDTs of Bioline and CTK and the ELISAs. (DOCX)

S7 Table Results for performance testing for the RDTs of Bioline and CTK and the ELISAs with n = 150. (DOCX)

S8 Table Results for the sensitivity of RDTs (Bioline and CTK) and the ELISA stratified by serotype. (DOCX)

S1 Fig Distribution of RDT results according to their Cq value. (DOCX)

S2 Fig Comparison of the Cq value results of serotype-specific dengue PCR. (DOCX)
