# Supplementary material for: Assessing the diagnostic performance of clinical, serological and molecular approaches to improve dengue case detection in the Peruvian Amazon
Source: PLoS Negl Trop Dis. 2026 Feb 9;20(2):e0013984. doi: 10.1371/journal.pntd.0013984 (PMC12928578; doi:10.1371/journal.pntd.0013984)
Supplement: S1 Table — (DOCX) [file pntd.0013984.s001.docx]

| **PCR** | **Oligo** | **Sequence (5’ → 3’)** | **Conc**  (µM) | **Ref.** |
| --- | --- | --- | --- | --- |
| **ZIKV** | ZIKV_For | CAGACTGCGACAGTTCGAG | 0.3 | (1) |
|  | ZIKV_Rev | AGAAACTCTCGYTTCCAAATCC | 0.3 |  |
|  | ZIKV_Probe | /56-FAM/CCTGTTGAT/ZEN/ACTGTTGYTAGCTYTCGCTTC/3IABkFQ/ | 0.1 |  |
| **CHIKV** | ChikSII | CCGACTCAACCATCCTGGAT | 0.4 | (2) |
|  | ChikAsII | GCAGACGCAGTGGTACTTCCT | 0.4 |  |
|  | ChikSI | TGATCCCGACTCAACCATCCT | 0.4 |  |
|  | ChikAsI | GGCAAACGCAGTGGTACTTCCT | 0.4 |  |
|  | Chik Probe | /5HEX/TCCGACATC/ZEN/ATCCTCCTTGCTGGC/3IABkFQ/ | 0.2 |  |
| **YFV** | YFV For | CTAATTGAGGTGYATTGGTCTG | 0.4 | (3, 4)  adapted |
|  | YFV Rev | CTGCTAATCGCTCAAMGAACG | 0.4 |  |
|  | YFV Probe | /5Cy5/ATCGAGTTG/TAO/CTAGGCAATAAACAC/3IAbRQSp/ | 0.2 |  |
| **DENV1** | D1 For | CAAAAGGAAGTCGTGCAATA | 0.2 | (5) |
|  | D1 Rev | CTGAGTGAATTCTCTCTACTGAACC | 0.2 |  |
|  | D1 Probe | /5TEX615/CATGTGGTTGGGAGCACGC/3IAbRQSp/ | 0.1 |  |
| **DENV2** | D2 For | CAGGCTATGGCACYGTCACGAT | 0.2 | (5) |
|  | D2 Rev | CCATYTGCAGCARCACCATCTC | 0.2 |  |
|  | D2 Probe | /5TEX615/CTCTCCRAGAACGGGCCTCGACTTCAA/3IAbRQSp/ | 0.1 |  |
| **DENV3** | D3 For | GGACTGGACACACGCACCCA | 0.2 | (5) |
|  | D3 Rev | CATGTCTCTACCTTCTCGACTTGYCT | 0.2 |  |
|  | D3 Probe | /5TEX615/ACCTGGATGTCGGCTGAAGGAGCTTG/3IAbRQSp/ | 0.1 |  |
| **Pan-DENV** | pDENV For | GCATATTGACGCTGGGARAG | 0.4 | (6, 7)  adapted |
|  | pDENV Rev1 | CTGTGCCTGGAATGATGCTG | 0.4 |  |
|  | pDENV Rev2 | CGCTCTGTGCCTGGATTGAT | 0.4 |  |
|  | pDENV Probe | /5TEX615/CCA + GA + GAT + CCT + GCT/3IAbRQSp/ | 0.2 |  |

**S1 Table:** **Primers and probes of the ZYDC-PCR.** Sequences, concentrations and the references of the primers and probes used in the ZYDC-multiplex PCR.

References

1. Chan JF-W, Yip CC-Y, Tee K-M, Zhu Z, Tsang JO-L, Chik KK-H, Tsang TG-W, Chan CC-S, Poon VK-M, Sridhar S, Yin F, Hung IF-N, Chau SK-Y, Zhang AJ, Chan K-H, Yuen K-Y. 2017. Improved detection of Zika virus RNA in human and animal specimens by a novel, highly sensitive and specific real-time RT-PCR assay targeting the 5'-untranslated region of Zika virus. Trop Med Int Health 22:594–603. doi:10.1111/tmi.12857.

2. Panning M, Grywna K, van Esbroeck M, Emmerich P, Drosten C. 2008. Chikungunya Fever in Travelers returning to Europe from the Indian Ocean Region, 2006. Emerg Infect Dis Vol. 14:416–422.

3. Domingo C, Patel P, Yillah J, Weidmann M, Méndez JA, Nakouné ER, Niedrig M. 2012. Advanced yellow fever virus genome detection in point-of-care facilities and reference laboratories. J Clin Microbiol 50:4054–4060. doi:10.1128/JCM.01799-12.

4. Méndez MC, Domingo C, Tenorio A, Pardo LC, Rey GJ, Méndez JA. 2012. Desarrollo de un método de transcripción reversa seguida de reacción en cadena de la polimerasa para la detección del virus de la fiebre amarilla. biomedica 33. doi:10.7705/biomedica.v33i0.1452.

5. Johnson BW, Russell BJ, Lanciotti RS. 2005. Serotype-Specific Detection of Dengue Viruses in a Fourplex Real-Time Reverse Transcriptase PCR Assay. J Clin Microbiol 43:4977–4983. doi:10.1128/JCM.43.10.4977–4983.2005.

6. Giry C, Roquebert B, Li-Pat-Yuen G, Gasque P, Jaffar-Bandjee M-C. 2017. Simultaneous detection of chikungunya virus, dengue virus and human pathogenic Leptospira genomes using a multiplex TaqMan® assay. BMC Microbiol 17:105. doi:10.1186/s12866-017-1019-1.

7. Alm E, Lesko B, Lindegren G, Ahlm C, Söderholm S, Falk KI, Lagerqvist N, Armstrong PM. 2014. Universal Single-Probe RT-PCR Assay for Diagnosis of Dengue Virus Infections. PLoS Negl Trop Dis 8:1–10. doi:10.1371/journal.pntd.0003416.
