# Supplementary material for: Assessing the diagnostic performance of clinical, serological and molecular approaches to improve dengue case detection in the Peruvian Amazon
Source: PLoS Negl Trop Dis. 2026 Feb 9;20(2):e0013984. doi: 10.1371/journal.pntd.0013984 (PMC12928578; doi:10.1371/journal.pntd.0013984)
Supplement: S2 Table — (DOCX) [file pntd.0013984.s002.docx]

| **PCR** | **Oligo** | **Sequence (5’ → 3’)** | **Conc**  (µM) | **Ref.** |
| --- | --- | --- | --- | --- |
| **Pan**  **DENV** | DF | AGGACYAGAGGTTAGAGGAGA | 20 | (1) |
|  | DR | CGYTCTGTGCCTGGAWTGAT | 20 |  |
|  | DP | /C-FAM/ACAGCATATTGACGCTGGGARAGACC/TAMRA/ | 10 |  |
| **ZIKV** | ZIKV 1086 | CCGCTGCCCAACACAAG | 20 | (2) |
|  | ZIKV 1162c | CCACTAACGTTCTTTTGCAGACAT | 20 |  |
|  | ZIKV 1107 | /HEX/AGCCTACCT/ZEN/TGACAAGCAGTCAGACACTCAA/ 3lABkFQ/ | 10 |  |
| **CHIKV** | F-CHIK | AAGCTYCGCGTCCTTTACCAAG | 20 | (3) |
|  | R-CHIK | CCAAATTGTCCYGGTCTTCCT | 20 |  |
|  | P-CHIK | /Cy5/CCAATGTCY/TAO/TCMGCCTGGACACCTTT/ 3lABkFQ | 10 |  |
|  |  |  |  |  |
| **DENV1** | D1 For | CAAAAGGAAGTCGTGCAATA | 1.0 | (4) |
|  | D1 Rev | CTGAGTGAATTCTCTCTACTGAACC | 1.0 |  |
|  | D1 Probe | /FAM/CATGTGGTTGGGAGCACGC/3IAbRQSp/ | 0.18 |  |
| **DENV2** | D2 For | CAGGCTATGGCACYGTCACGAT | 0.5 | (4) |
|  | D2 Rev | CCATYTGCAGCARCACCATCTC | 0.5 |  |
|  | D2 Probe | /HEX/CTCTCCRAGAACGGGCCTCGACTTCAA/3IAbRQSp/ | 0.18 |  |
| **DENV3** | D3 For | GGACTGGACACACGCACCCA | 1.0 | (4) |
|  | D3 Rev | CATGTCTCTACCTTCTCGACTTGYCT | 1.0 |  |
|  | D3 Probe | /TexRed/ACCTGGATGTCGGCTGAAGGAGCTTG/3IAbRQSp/ | 0.18 |  |
| **DENV4** | D4 For | TTGTCCTAATGATGCTRGTCG | 0.5 | (4) |
|  | D4 Rev | TCCACCYGAGACTCCTTCCA | 0.5 |  |
|  | D4 Probe | /Cy5/TYCCTACYCCTACGCATCGCATTCCG/3IAbRQSp/ | 0.18 |  |

**S2 Table:** **Primers and probes of the ZDC-PCR and the reference PCR.** Sequences, concentrations and the references of the primers and probes used in the triplex-PCR (ZDC-PCR) and the serotype specific dengue PCR (DENV1-DENV4).

References

1. Leparc-Goffart I, Baragatti M, Temmam S, Tuiskunen A, Moureau G, Charrel R, Lamballerie X de. 2009. Development and validation of real-time one-step reverse transcription-PCR for the detection and typing of dengue viruses. J Clin Virol 45:61–66. doi:10.1016/j.jcv.2009.02.010.

2. Lanciotti RS, Kosoy OL, Laven JJ, Velez JO, Lambert AJ, Johnson AJ, Stanfield SM, Duffy MR. 2008. Genetic and serologic properties of Zika virus associated with an epidemic, Yap State, Micronesia, 2007. Emerg Infect Dis 14:1232–1239. doi:10.3201/eid1408.080287.

3. Pastorino B, Bessaud M, Grandadam M, Murri S, Tolou HJ, Peyrefitte CN. 2005. Development of a TaqMan RT-PCR assay without RNA extraction step for the detection and quantification of African Chikungunya viruses. J Virol Methods 124:65–71. doi:10.1016/j.jviromet.2004.11.002.

4. Johnson BW, Russell BJ, Lanciotti RS. 2005. Serotype-Specific Detection of Dengue Viruses in a Fourplex Real-Time Reverse Transcriptase PCR Assay. J Clin Microbiol 43:4977–4983. doi:10.1128/JCM.43.10.4977–4983.2005.
