# Supplementary material for: Assessing the diagnostic performance of clinical, serological and molecular approaches to improve dengue case detection in the Peruvian Amazon
Source: PLoS Negl Trop Dis. 2026 Feb 9;20(2):e0013984. doi: 10.1371/journal.pntd.0013984 (PMC12928578; doi:10.1371/journal.pntd.0013984)
Supplement: S3 Table — (DOCX) [file pntd.0013984.s003.docx]

| Group | All serotypes | DENV1 | DENV2 | DENV3 |
| --- | --- | --- | --- | --- |
| DwoWS (n, %)  Median Cq  (IQR) | 56 (66.7%) | 11 (19.6%) | 30 (53.6%) | 15 (26.8%) |
|  | 20.2  (18.0-23.8) | 18.4  (14.6-21.0) | 20.2  (18.9-23.3) | 21.8  (18.6-30.4) |
| DwWS (n, %)  Median Cq  (IQR) | 28 (33.3%) | 3 (10.7%) | 17 (60.7%) | 8 (28.6%) |
|  | 26.9  (21.4-33.9) | 15.1  (13.5-23.7) | 27.2  (21.6-33.9) | 27.4  (22.0-34.6) |
| p-value | **0.009** | 0.769 | **0.007** | 0.506 |

**S3 Table:** **Distribution of PCR-confirmed DENV infections** **with and without warning signs and their median Cq values.** DwoWS = dengue without warning signs; DwWS = dengue with warning signs. The table shows the number of PCR-confirmed DENV infections and their corresponding Cq values stratified by WHO 2009 clinical classification and serotype. The bottom row shows p-values from Wilcoxon rank-sum tests comparing Cq values between DwWS and DwoWS within each serotype and overall. Numbers differ from the total PCR-confirmed DENV cases because some patients lacked WHO clinical classification data.
