# Supplementary material for: Assessing the diagnostic performance of clinical, serological and molecular approaches to improve dengue case detection in the Peruvian Amazon
Source: PLoS Negl Trop Dis. 2026 Feb 9;20(2):e0013984. doi: 10.1371/journal.pntd.0013984 (PMC12928578; doi:10.1371/journal.pntd.0013984)
Supplement: S4 Table — (DOCX) [file pntd.0013984.s004.docx]

| **Predictor** | **Adjusted OR (95% CI)** | **p-value** |
| --- | --- | --- |
| DENV 2 vs DENV1 | 2.3 (0.52–10.27) | 0.303 |
| DENV 3 vs DENV1 | 1.55 (0.26–9.17) | 0.635 |
| Age (years) | 1.01 (0.97–1.06) | 0.670 |
| Male sex | 0.48 (0.20–1.17) | 0.226 |
| DASO | 2.02 (1.43–2.85) | <0.001 |

**S4 Table: Multivariable logistic regression evaluating serotype as a predictor of dengue with warning signs (DwWS).** The model adjusts for age, sex, and days after symptom onset (DASO). DENV1 is the reference category.
