# Supplementary material for: Assessing the diagnostic performance of clinical, serological and molecular approaches to improve dengue case detection in the Peruvian Amazon
Source: PLoS Negl Trop Dis. 2026 Feb 9;20(2):e0013984. doi: 10.1371/journal.pntd.0013984 (PMC12928578; doi:10.1371/journal.pntd.0013984)
Supplement: S5 Table — (DOCX) [file pntd.0013984.s005.docx]

| **DENV** | **DENV pos** | **Median Cq value** | **∆Cq vs DENV1** | **p-value** |
| --- | --- | --- | --- | --- |
| Overall | 88 (32.6%) | 21.6 (IQR: 18.5-31.1) |  |  |
| DENV1* | 15 (17.1%) | 18.4 (IQR: 14.5-23.5) | reference |  |
| DENV2* | 49 (55.7%) | 21.5 (IQR: 19.0-27.2) | +4.29 | 0.031 |
| DENV3* | 24 (27.3%) | 23.0 (IQR: 19.8-34.6) | +5.85 | 0.009 |

**S5 Table: Distribution of DENV positive samples with their median Cq value for the reference PCR**. *Percentages are calculated based on the total number of DENV positive samples. Number and proportion of DENV positive samples, the median Cq values (IQR) for all DENV positive samples and stratified by DENV1, DENV2 and DENV3, and the results of a linear regression model evaluating serotype as a predictor of Cq, adjusted for days after symptom onset. Adjusted differences exceed the crude differences in median Cq values because the model controls for variation in DASO, which strongly influences viral load, thus estimating the independent effect of serotype.
