# Supplementary material for: Assessing the diagnostic performance of clinical, serological and molecular approaches to improve dengue case detection in the Peruvian Amazon
Source: PLoS Negl Trop Dis. 2026 Feb 9;20(2):e0013984. doi: 10.1371/journal.pntd.0013984 (PMC12928578; doi:10.1371/journal.pntd.0013984)
Supplement: S6 Table — (DOCX) [file pntd.0013984.s006.docx]

| N of test |  | SE | SP | Accuracy | Kappa |
| --- | --- | --- | --- | --- | --- |
| N=270 | NS1 Bioline | **0,720**  (95% CI:  0.610-0.809) | **0.964**  (95% CI:  0.928-0.983) | **0.896**  (95% CI:  0.855-0.927) | 0.726 |
|  | IgM Bioline | **0.467**  (95% CI:  0.358-0.578) | **0.610**  (95% CI:  0.540-0.676) | **0.570**  (95% CI:  0.511-0.628) | 0.067 |
|  | NS1/IgM Bioline | **0.880**  (95% CI:  0.787-0.936) | **0.590**  (95% CI:  0.520-0.656) | **0.670**  (95% CI:  0.612-0.724) | 0.364 |
| N=257 | NS1 CTK | **0.726**  (95% CI:  0.614-0.815) | **0.962**  (95% CI:  0.924-0.981) | **0.895**  (95% CI:  0.851-0.927) | 0.727 |
|  | IgM CTK | **0.151**  (95% CI:  0.086-0.250) | **0.864**  (95% CI:  0.807-0.906) | **0.661**  (95% CI:  0.602-0.717) | 0.017 |
|  | NS1/IgM CTK | **0.753**  (95% CI:  0.644-0.838) | **0.832**  (95% CI:  0.771-0.879) | **0.809**  (95% CI:  0.757-0.853) | 0.555 |
| N=151 | NS1_ELISA | **0.729**  (95% CI:  0.590-0.834) | **0.942**  (95% CI:  0.879-0.973) | **0.874**  (95% CI:  0.812-0.918) | 0.698 |
| N=35 | IgM_ELISA | **0.333**  (95% CI:  0.097-0.700) | **0.931**  (95% CI:  0.780-0.981) | **0.829**  (95% CI:  0.673-0.919) | 0.305 |

**S6 Table:** **Results for performance testing for the RDTs of Bioline and CTK and the ELISAs**. The results were compared to the ZYDC-PCR for all samples with the number of samples with results available for each test.
