# Supplementary material for: Assessing the diagnostic performance of clinical, serological and molecular approaches to improve dengue case detection in the Peruvian Amazon
Source: PLoS Negl Trop Dis. 2026 Feb 9;20(2):e0013984. doi: 10.1371/journal.pntd.0013984 (PMC12928578; doi:10.1371/journal.pntd.0013984)
Supplement: S7 Table — (DOCX) [file pntd.0013984.s007.docx]

| N=149 | SE | SP | Accuracy | Kappa |
| --- | --- | --- | --- | --- |
| NS1 Bioline | **0.792**  (95%CI:  0.657-0.883) | **0.941**  (95% CI: 0.876-0.972) | **0.893**  (95% CI: 0.833-0.933) | 0.749 |
| IgM Bioline | **0.417**  (95% CI:  0.288-0.557) | **0.574**  (95% CI: 0.4.7-0.666) | **0.523**  (95% CI: 0.444-0.602) | -0.008 |
| NS1/IgM Bioline | **0.896**  (95% CI:  0.778-0.955) | **0.535**  (95% CI: 0.438-0.629) | **0.651**  (95% CI: 0.572-0.723) | 0.350 |
| NS1 CTK | **0.792**  (95% CI:  0.657-0.883) | **0.941**  (95% CI: 0.876-0.972) | **0.893**  (95% CI: 0.833-0.933) | 0.749 |
| IgM CTK | **0.083**  (95% CI:  0.033-0.196) | **0.891**  (95% CI: 0.815-0.938) | **0.631**  (95% CI: 0.551-0.704) | -0.031 |
| NS1/IgM CTK | **0.792**  (95% CI:  0.657-0.883) | **0.832**  (95% CI: 0.747-0.892) | **0.819**  (95% CI: 0.749-0.872) | 0.600 |
| NS1 ELISA | **0.729**  (95% CI:  0.590-0.834) | **0.941**  (95% CI: 0.876-0.972) | **0.872**  (95% CI: 0.809-0.917) | 0.696 |

**S7 Table:** **Results for performance testing for the RDTs of Bioline and CTK and the ELISAs with n=149**. The results compared to the ZYDC-PCR for the number of samples for which results where available for all tests (n=149). SE: sensitivity, SP: specificity.
