# Supplementary material for: Assessing the diagnostic performance of clinical, serological and molecular approaches to improve dengue case detection in the Peruvian Amazon
Source: PLoS Negl Trop Dis. 2026 Feb 9;20(2):e0013984. doi: 10.1371/journal.pntd.0013984 (PMC12928578; doi:10.1371/journal.pntd.0013984)
Supplement: S8 Table — (DOCX) [file pntd.0013984.s008.docx]

|  | | **Sensitivity** | | |
| --- | --- | --- | --- | --- |
| Bioline  (n=270) | Serotype | **DENV1 (n=15)** | **DENV2 (n=49)** | **DENV3 (n=24)** |
|  | NS1 | **0.750**  (95% CI: 0.468-0.911) | **0.689**  (95% CI: 0.543-0.805) | **0.778**  (95% CI: 0.548-0.910) |
|  | IgM | 0.500  (95% CI: 0.254-0.746) | 0.489  (95% CI: 0.350-0.630) | 0.389  (95% CI: 0.203-0.614) |
|  | NS1&IgM | **1.000**  (95% CI: 0.758-1.000 | **0.867**  (95% CI: 0.738-0.937) | **0.833**  (95% CI: 0.608-0.942) |
| CTK  (n=257) | **Serotype** | **DENV1 (n=15)** | **DENV2 (n=48)** | **DENV3 (n=23)** |
|  | NS1 | **0.750**  (95% CI: 0.468-0.911) | **0.727**  (95% CI: 0.582-0.837) | **0.706**  (95% CI: 0.469-0.867) |
|  | IgM | **0.167**  (95% CI: 0.047-0.448) | **0.136**  (95% CI: 0.064-0.267) | **0.176**  (95% CI: 0.062-0.410) |
|  | NS1&IgM | **0.750**  (95% CI: 0.468-0.911) | **0.750**  (95% CI: 0.606-0.854) | **0.765**  (95% CI: 0.527-0.904) |
| ELISA  (n=151) | **Serotype** | **DENV1 (n=7)** | **DENV2 (n=32)** | **DENV3 (n=16)** |
|  | NS1 | **0.667**  (95% CI: 0.300-0.903) | **0.690**  (95% CI: 0.508-0.827) | **0.846**  (95% CI: 0.578-0.957) |

**S8 Table:** **Results for the sensitivity of RDTs (Bioline and CTK) and the ELISA stratified by serotype.**
