# Supplementary material for: Assessing the diagnostic performance of clinical, serological and molecular approaches to improve dengue case detection in the Peruvian Amazon
Source: PLoS Negl Trop Dis. 2026 Feb 9;20(2):e0013984. doi: 10.1371/journal.pntd.0013984 (PMC12928578; doi:10.1371/journal.pntd.0013984)
Supplement: S1 Fig — (DOCX) [file pntd.0013984.s009.docx]

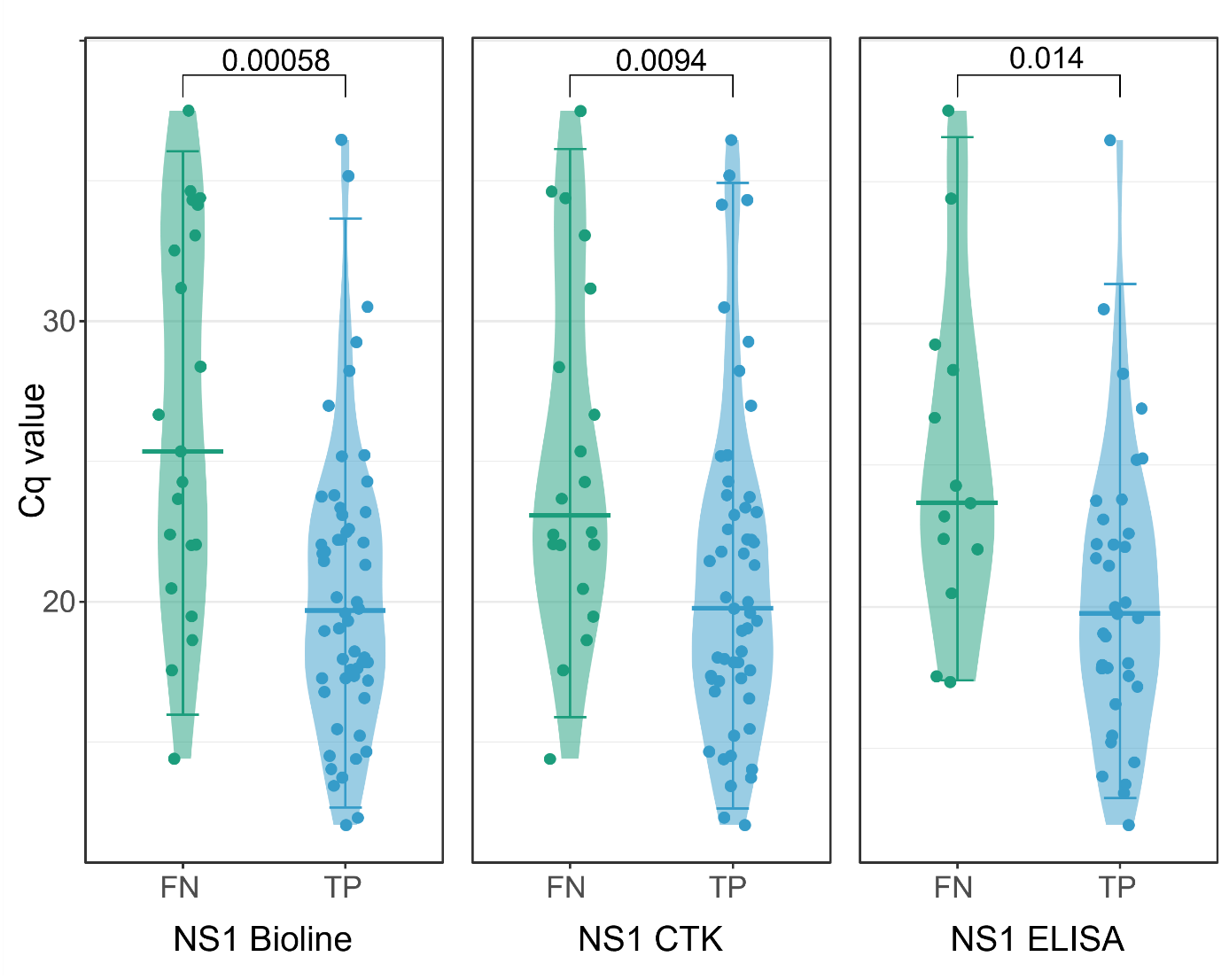


**S1 Fig:** **Distribution of RDT results according to their Cq value**. Violin plots showing the distribution of RDT results stratified by the Cq values obtained with ZYDC-PCR. Each dot represents an individual sample. FN stands for false negative and TP for true positive.
