# Supplementary material for: Assessing the diagnostic performance of clinical, serological and molecular approaches to improve dengue case detection in the Peruvian Amazon
Source: PLoS Negl Trop Dis. 2026 Feb 9;20(2):e0013984. doi: 10.1371/journal.pntd.0013984 (PMC12928578; doi:10.1371/journal.pntd.0013984)
Supplement: S2 Fig — (DOCX) [file pntd.0013984.s010.docx]

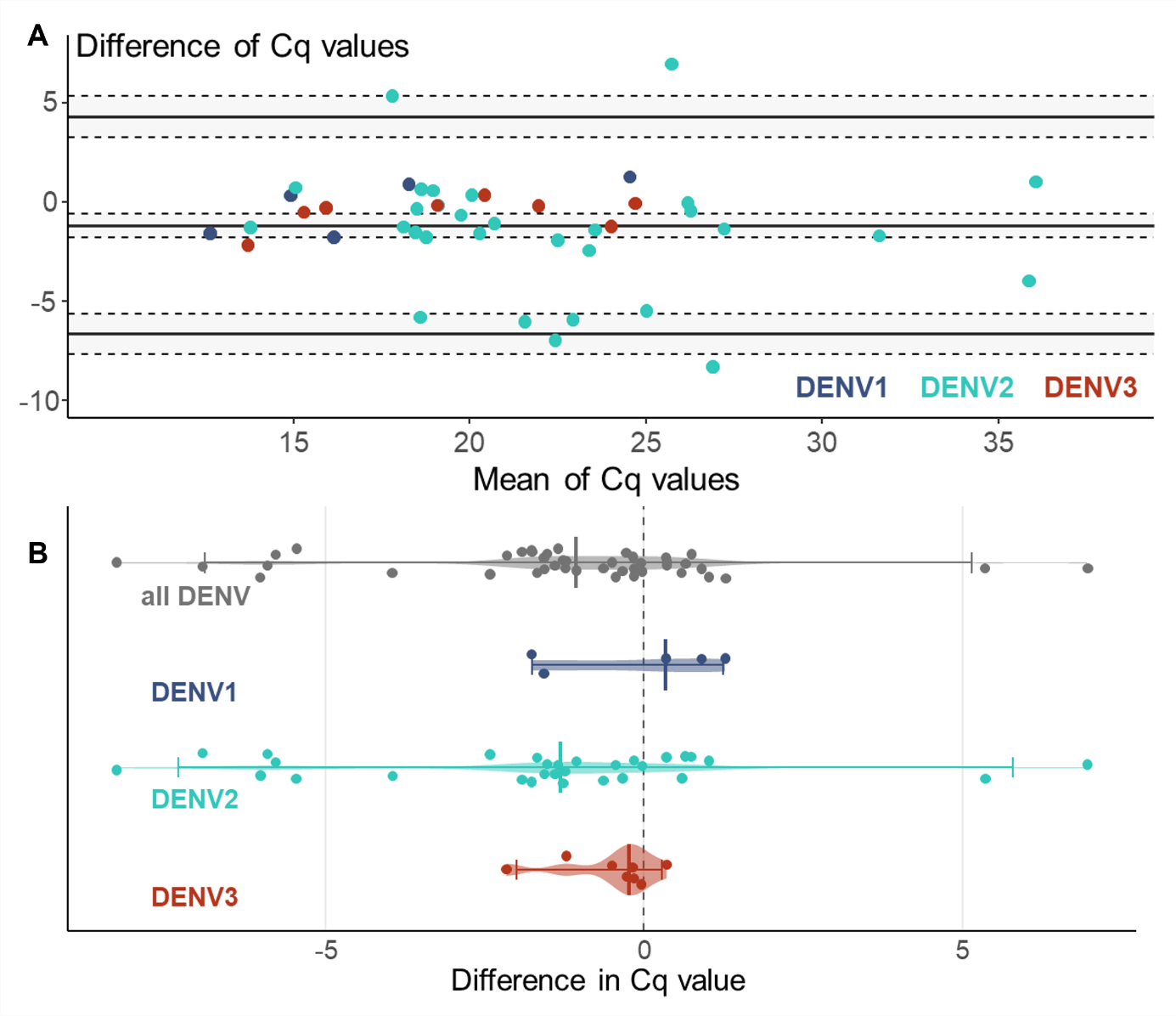


**S2 Fig:** **Comparison of the Cq value results of serotype-specific dengue PCR**. A subset of dengue positive RNA extracted in Yurimaguas (n=44) was run with the serotype-specific dengue PCR and compared to the results of the serotype-specific dengue PCR with RNA extracted in Lima with A) Bland-Altman plot and B) Violinplot, stratified by dengue serotype.
